# Considerations for maximizing the adaptive potential of restored coral populations in the western Atlantic

**DOI:** 10.1002/eap.1978

**Published:** 2019-08-19

**Authors:** Iliana B. Baums, Andrew C. Baker, Sarah W. Davies, Andréa G. Grottoli, Carly D. Kenkel, Sheila A. Kitchen, Ilsa B. Kuffner, Todd C. LaJeunesse, Mikhail V. Matz, Margaret W. Miller, John E. Parkinson, Andrew A. Shantz

**Affiliations:** ^1^ Department of Biology Pennsylvania State University University Park Pennsylvania 16803 USA; ^2^ Department of Marine Biology and Ecology Rosenstiel School of Marine and Atmospheric Science University of Miami Miami Florida 33149 USA; ^3^ Department of Biology Boston University Boston Massachusetts 02215 USA; ^4^ School of Earth Sciences Ohio State University Columbus Ohio 43210 USA; ^5^ Department of Biological Sciences University of Southern California Los Angeles California 90007 USA; ^6^ U.S. Geological Survey 600 4th Street S. St. Petersburg Florida 33701 USA; ^7^ Department of Integrative Biology The University of Texas at Austin Austin Texas 78712 USA; ^8^ SECORE International Miami Florida 33145 USA; ^9^ Department of Integrative Biology University of South Florida Tampa Florida 33620 USA

**Keywords:** adaptive potential, assisted gene flow, biomarkers, coral restoration, genetic diversity, inbreeding, outbreeding, phenotypic resilience, population enhancement, species selection, unintended selection

## Abstract

Active coral restoration typically involves two interventions: crossing gametes to facilitate sexual larval propagation; and fragmenting, growing, and outplanting adult colonies to enhance asexual propagation. From an evolutionary perspective, the goal of these efforts is to establish self‐sustaining, sexually reproducing coral populations that have sufficient genetic and phenotypic variation to adapt to changing environments. Here, we provide concrete guidelines to help restoration practitioners meet this goal for most Caribbean species of interest. To enable the persistence of coral populations exposed to severe selection pressure from many stressors, a mixed provenance strategy is suggested: genetically unique colonies (genets) should be sourced both locally as well as from more distant, environmentally distinct sites. Sourcing three to four genets per reef along environmental gradients should be sufficient to capture a majority of intraspecies genetic diversity. It is best for practitioners to propagate genets with one or more phenotypic traits that are predicted to be valuable in the future, such as low partial mortality, high wound healing rate, high skeletal growth rate, bleaching resilience, infectious disease resilience, and high sexual reproductive output. Some effort should also be reserved for underperforming genets because colonies that grow poorly in nurseries sometimes thrive once returned to the reef and may harbor genetic variants with as yet unrecognized value. Outplants should be clustered in groups of four to six genets to enable successful fertilization upon maturation. Current evidence indicates that translocating genets among distant reefs is unlikely to be problematic from a population genetic perspective but will likely provide substantial adaptive benefits. Similarly, inbreeding depression is not a concern given that current practices only raise first‐generation offspring. Thus, proceeding with the proposed management strategies even in the absence of a detailed population genetic analysis of the focal species at sites targeted for restoration is the best course of action. These basic guidelines should help maximize the adaptive potential of reef‐building corals facing a rapidly changing environment.

## Introduction

Coral reef ecosystems face unparalleled destruction due to anthropogenic climate change (McClenachan et al. 2017, Hughes et al. 2018), and protective coping mechanisms observed in extant coral populations may be overwhelmed by the rate of ocean temperature change (Ainsworth et al. 2016). Thus, the recovery of reef ecosystems hinges on immediate and decisive actions to reduce CO_2_ emissions globally. Meanwhile, large investments in coral reef restoration and the population enhancement of critical reef‐building species have been made in an effort to revive coral communities and maintain some ecosystem function and services (Ladd et al. 2018). Technologies such as artificial selection or genetic engineering are being contemplated to “design” coral populations capable of withstanding current and future environmental challenges. However, field applications of these technologies are not yet ready (van Oppen et al. 2001, Torda et al. 2014, Cleves et al. 2018). Challenges include our rudimentary understanding of the genetic basis of traits that will be essential in the future and limited knowledge of their interactions (i.e., trade‐offs; Muller et al. 2018), as well as logistical difficulties of propagating and outplanting engineered corals at ecologically significant scales. Protocols for assessing the risks associated with such interventions are being developed but have yet to be widely adopted (Baums 2008, IUCN/SSC 2013).

We contend that currently the best approach to improve reef resilience via direct, restoration‐focused intervention is to harness and foster the adaptive genetic diversity that is already present in coral populations while efforts to reduce greenhouse gas emissions continue (Kleypas et al. 2016, Bay et al. 2017, Matz et al. 2018). Every coral species exists across a variety of environmental gradients, some of which occur over small spatial scales (e.g., fore, back, and patch reefs) while others occur over very large ones (e.g., across ocean‐basins). There is mounting evidence that corals genetically adapt to their local conditions (Polato et al. 2010, Barshis et al. 2013, Kenkel et al. 2013, Dixon et al. 2015), so each species is in fact a system of sub‐populations adapted to diverse environments and connected by larval migration. In linked sets of metapopulations, global change could be rapidly matched by the recombination of pre‐existing adaptive genetic variants, i.e., adaptation based on standing genetic variation (Hermisson and Pennings 2005, Baums 2008, Whiteley et al. 2011). Increasingly severe and frequent mortality events have already led to widespread and continued loss of coral cover (Eakin et al. 2010, Smith et al. 2013, Precht et al. 2016, Walton et al. 2002) and exerted strong selection pressure on these metapopulations. Therefore, adaptation is likely to be rapid: survivors of high mortality events are particularly robust and may be expected to eventually spawn a new generation of better‐adapted corals (Libro and Vollmer 2016, Muller et al. 2018). However, reduced connectivity between these metapopulations, lower fertilization rates because colonies are spread further apart (i.e., allee effects; Knowlton 2001), and universally declining environmental conditions across most reef sites reduce the realization of this potential. If local genetic variation can be maintained, environmental conditions can be stabilized, and genetic exchange among populations can continue unimpeded, adaptation may allow corals to keep pace with climate change for the next 100 years or longer (Matz et al. 2018). In this context, many interventions are available now that can help maintain high local genetic variation and continued exchange among populations.

From this evolution‐centric perspective, the goal of restoration is to establish self‐sustaining, sexually reproducing coral populations thereby promoting continuous genetic adaptation of the species, both locally and throughout its range. This approach is congruent with endangered species recovery goals and aligned with but distinct from some current restoration guidelines focused on ecological goals such as coral cover or habitat provision. Measuring success using these metrics by themselves may be insufficient to ensure the long‐term future of restored corals, as ecological success may only be temporary in the absence of self‐sustaining coral populations. Instead, our focus is on providing practical guidelines to maintain the genetic diversity and phenotypic resilience required for corals to survive and produce genetically diverse and viable offspring that could serve as raw material for natural selection. Layering both evolutionary and ecological components will maximize the resilience of restored coral populations.

The need for concrete guidelines is particularly pressing in the Caribbean, where investments in active restoration of coral populations have occurred over the last two decades and continue to increase at a rate that exceeds similar efforts in other regions (Young et al. 2017, Chamberland et al. 2015, Lirman and Schopmeyer 2016). Caribbean reefs also tend to be highly degraded and suffer from long‐term recruitment failure of the two major reef‐building species, *Acropora palmata* (Williams et al. 2015) and *Orbicella faveolata* (Hughes and Tanner 2000), Although some reefs still feature substantial stands of these species, without sexual recruitment they may already be effectively extinct (Honnay and Bossuyt 2005). Most alarmingly, since there is no ecological redundancy in the Caribbean to replace these species in their reef‐accretion function, many Caribbean reefs have shifted from net accretional to net erosional states (Kuffner and Toth 2016, Yates et al. 2018). Shifts to net erosional states are now a global phenomenon (Perry et al. 2018), with concomitant increases in the vulnerability of coastal communities to inundation and shoreline erosion throughout the tropical world (Beck et al. 2018, Storlazzi et al. 2018). Although outplanting corals using nursery‐propagated stock can help restore some ecological functions (Montoya Maya et al. 2016) and may buy time to prevent regional extirpation, without sexual recombination and the shuffling of alleles to promote adaptation, the long‐term future of these corals appears bleak.

**Figure 1:**
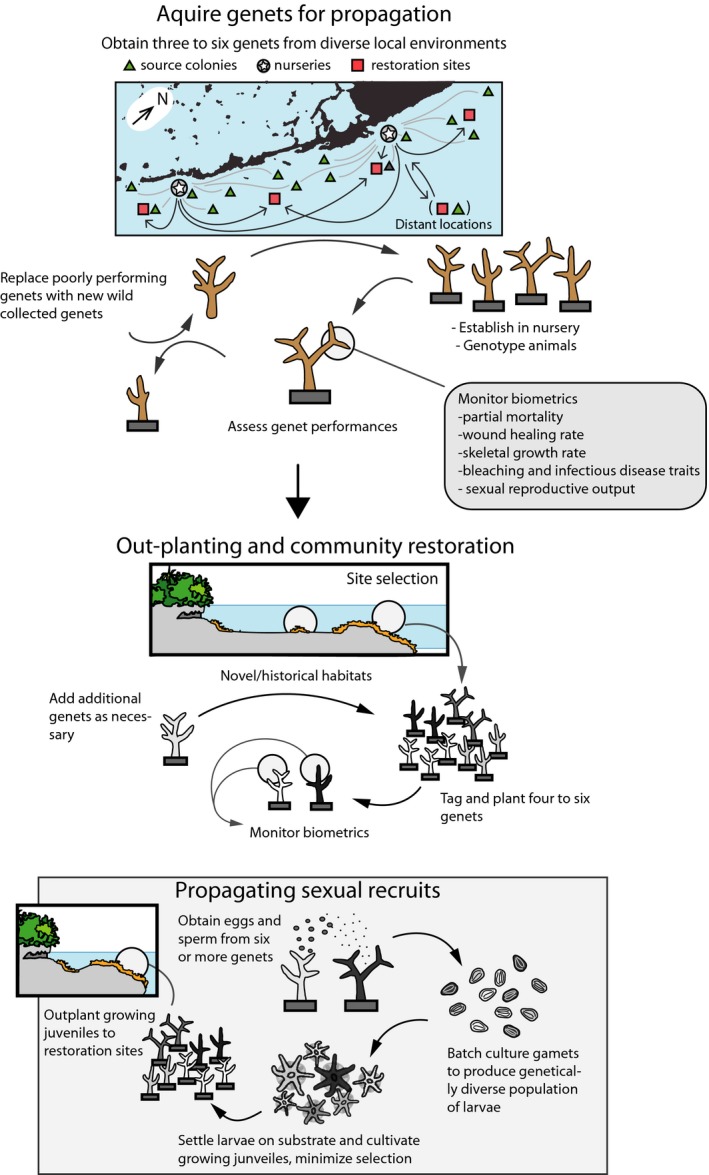
Overview of basic restoration guidelines to maximize the adaptive potential of reef‐building corals facing a rapidly changing environment. A genet is defined as a genetically unique colony or collection of colonies (ramets) that can trace their ancestry back to the same sexual reproductive event (i.e., they stem from the same settler and, hence, share the same genome).

The guidelines presented here aim to re‐establish populations that are capable of sexual recruitment and genetic exchange (Fig. 1). These include recommendations on how different reef habitats should be strategically sampled to capture much of the adaptive genetic variants existing in coral populations, and how sampled genets should be outplanted and monitored. We discuss possible enhancements to restoration practices such as routine genotyping of propagated stock, trait‐based assessment of genet performance, jump‐starting genetic admixture by producing first‐generation offspring for outplanting, and promoting long‐range genetic exchange (“assisted gene flow,” AGF). In addition, because corals host photosynthetic micro‐algae [family Symbiodiniaceae sensu LaJeunesse et al. (2018)], we also consider possibilities for the management of endosymbiont diversity in restored populations. Finally, we discuss the balance of risks and benefits associated with implementing the recommended strategies. The review is the product of the Coral Restoration Genetics Working group, one of the five original working groups of the Coral Restoration Consortium (Appendix S1: Section 1).

## Prioritizing Coral Species for Restoration Efforts

**Figure 2:**
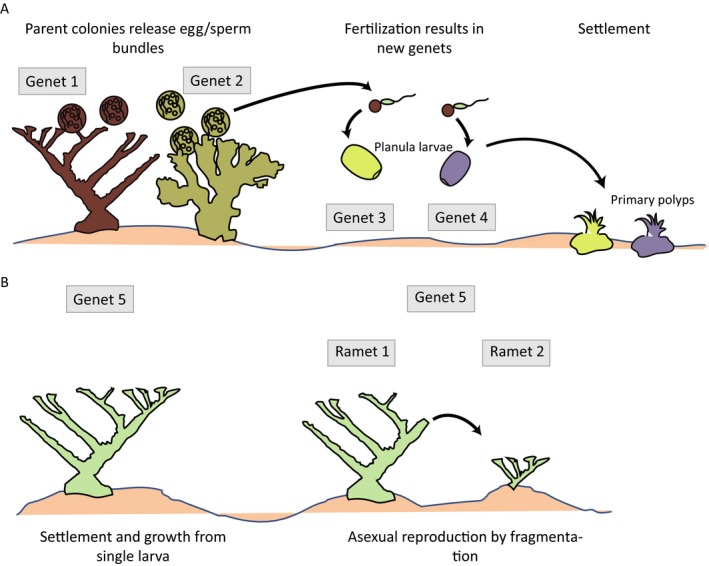
Coral reproduction. (A) Most reef‐building species in the Caribbean are self‐incompatible hermaphroditic broadcast spawners. Adult colonies release egg–sperm bundles that float to the surface where they break apart and mix with gametes from other colonies of the same species. After fertilization and development, larvae settle onto the reef, metamorphose into primary polyps, and grow into new genets. (B) Over time, genets may fragment. These fragments can reattach to form new ramets of the same genet. A genet is defined as a genetically unique colony or collection of colonies (ramets) that can trace their ancestry back to the same sexual reproductive event (i.e., they stem from the same settler and, hence, share the same genome). Adopted from Devlin‐Durante et al. (2016).

Coral species selection for restoration will depend upon the goals of the project, which could include shoreline protection, fisheries habitat provisioning, species conservation, tourism, or a combination thereof. Given that funding, nursery space, and time are limited, we propose that ecosystem‐based restoration efforts for Caribbean corals should prioritize species that are (1) foundation reef builders; (2) experiencing severe declines in cover (Appendix S2: Fig. S1); and (3) consistently failing to sexually recruit (Fig. 2). In the Caribbean and western north Atlantic, only a limited number of coral species build reef framework, the overall species diversity is lower, and functional redundancy is limited compared to the Indo‐Pacific (Bellwood et al. 2004), making species selection comparatively straightforward. There is a striking lack of sexual recruitment for many of the framework‐building species throughout the basin (Hughes and Tanner 2000, Edmunds and Elahi 2007, Williams et al. 2015, Davies et al. 2013a) and recruitment has shifted from long‐lived broadcast‐spawning species to more weedy brooding species (Rogers et al. 1984, Hughes and Tanner 2000, Green et al. 2008, but see Vermeij et al. 2014).

Current restoration efforts target *Acropora cervicornis*,* A. palmata*,* Orbicella faveolata*, and *O. annularis*. All are broadcast‐spawning species and are among those experiencing widespread population declines and sexual recruitment failure (van Woesik et al. 1993). *A. cervicornis* takes up the most space in current nurseries (Lirman and Schopmeyer 2016). On some reefs in Belize and the Dominican Republic, *A. cervicornis* has been restored to high densities (Lirman and Schopmeyer 2016) and outplants are spawning (Carne and Baums 2016). In Florida, a shift to *A. palmata* and multi‐species restoration with an emphasis on reef‐building is now underway. Additional efforts have been extended locally to species where extirpation might be imminent (i.e., *Dendrogyra cylindrus* in the Florida Keys). We suggest that efforts be expanded to target *Pseudodiploria* spp., *Siderastrea siderea*,* Stephanocoenia intersepta*,* Montastrea cavernosa*,* Colpophyllia natans*, and *Orbicella franksi*, as these species each meet two prioritization criteria (Appendix S2: Fig. S1), are already managed in some locations, and are good candidate species for rebuilding reef structure long‐term, in part because these species still successfully produce sexual recruits.

For coral taxa that are rare or not major reef builders (e.g., *Dendrogyra*,* Agaricia*,* Porites*,* Dichocoenia*,* Favia*), we propose strategic genetic banking as a complementary activity to active restoration (Hagedorn et al. 2012). This compromise will free up resources for major reef builders of immediate concern while safeguarding genetic material for future action on these rare members that increase community diversity and may play important, if unknown, roles in ecosystem processes (Bellwood et al. 2006). Thus, efficient cryo‐banking techniques should be developed for species whose gametes can be collected, and ex situ gene banking (i.e., keeping live animals in aquaria) might be considered for others. We also recommend that restoration feasibility studies be conducted for rare species experiencing massive population declines, multi‐year sexual recruitment failure, and those that exhibit unique life histories such as *Dendrogyra cylindrus* (Neely et al. 2018, Chan et al. 2019), to help identify and address challenges associated with restoring these species. Lastly, we propose that species prioritizations should follow an adaptive management strategy under which demographic monitoring of natural and outplanted populations is evaluated every 5–10 years to determine changes in population growth rates (λ) or sexual recruitment rates (which serve as a reasonable proxy when λ cannot be estimated). Sexual recruitment rates can be monitored via both recruitment tiles (Humanes and Bastidas 2015) and genotyping (Box 1; Tables 1, 2; Appendix S3: Section S1).

**Table 1:** An overview of different genotyping methods employed for Caribbean coral species and their symbionts

Species	Allozymes	AFLP	Msats	SNPs	References
Coral					
*Acropora cervicornis*			✓ (12)	✓	Baums et al. (2005a, 2009), Drury et al. (2016)
* Acropora palmata*	✓		✓ (13)	✓	Baums et al. (2005a, 2009), Devlin‐Durante and Baums (2017)
* Dendrogyra cylindrus*			✓ (11)		Chan et al. (2019)
* Favia fragum*			✓ (15)		Carlon and LippE (2008)
* Orbicella annularis*	✓	✓	✓ (14)	✓	Van Veghel and Bak (2011), Lopez et al. (1999), Fukami et al. (2004), Severance et al. (2004), Davies et al. (2013b), Prada et al. (2016)
* Orbicella faveolata*		✓	✓ (9)	✓	Fukami et al. (2004), Lopez et al. (1999), Davies et al. (2013b), Prada et al. (2016)
* Montastrea cavernosa*		✓	✓ (14)		Shearer and Coffroth (2004), Brazeau et al. (2013), Serrano et al. (2014), Jarett et al. (2017)
* Porites astreoides*	✓	✓	✓ (15)		Weil (2018), Brazeau et al. (1998), Shearer and Coffroth (2004), Kenkel et al. (2013), Serrano et al. (2016)
Symbiont					
* Symbiodinium microadriaticum* (ITS2 type A1)	✓				Schoenberg and Trench (1980)
* S. “fitti”* (ITS2 type A3)			✓ (13)		Baillie et al. (2000a, b), Pinzón et al. (2011), Baums et al. (2014a)
* Breviolum “dendrogyrum”* (ITS2 type B1)			✓ (7)		Santos and Coffroth (2003), Pettay and LaJeunesse (2007), Chan et al. (2019)
* B. endomadracis* (ITS2 type B7)			✓ (3)		Santos and Coffroth (2003), Pettay and LaJeunesse (2007)
* B. minutum* (ITS2 type B1)			✓ (3)		Santos and Coffroth (2003), Pettay and LaJeunesse (2007)
* B. psygmophilum* (ITS2 type B2)			✓ (6)		Pettay and LaJeunesse (2007), Grupstra et al. (2017)
* Durusdinium trenchii* (ITS2 type D1a)			✓ (17)		Pettay and LaJeunesse (2009), Wham et al. (2001)

*Notes:*

AFLP, amplification fragment length polymorphism; Msats, microsatellites; SNPs, single nucleotide polymorphisms. A ✓ indicates that the method was used for that species. Numbers in parenthesis give the number of microsatellite loci available.

**Table 2:** Comparison of the microsatellites and SNP genotyping methods

Measure	Msats	SNP‐based methods				
		Targeted‐enrichment capture	Microarray	Microfluidics	Traditional GBS/RAD‐tag	RAD capture
No. markers	10^1^	10^2^	10^3^–10^5^	10^2^	10^3^–10^5^	10^2^–10^3^
Minimum no. samples	1	6	96	96	1	1
Sample preparation^a^	moderate	moderate	low‐moderate	low	low‐moderate	low‐ moderate
Technical expertise^b^	moderate	high	moderate	moderate‐high	high	moderate
Computational resources^c^	low	high	moderate	moderate	high	moderate
Reproducible between labs^d^	moderate	high	high	high	high	high
Estimated price per sample	US$50	US$450	US$50	US$10	US$75	US$50–70

a Low, library preparation and sequencing can be completed at a core‐facility provider; moderate, multiple‐day process but limited hands‐on time.

b Based on training requirements. Low, minimal training; moderate, some advanced training; high, highly advanced training (Grover and Sharma 2016).

c Based on the computational resource demands. Low, analyzed on a standard computer with minimal storage; moderate, analyzed on standard computer with large data storage requirements; high, analyzed on high‐performance computer with large data storage.

d Sensitivity of the method to differences between laboratories or sequencing facilities. Moderate, analysis can be impacted by laboratory conditions (e.g., different PCR buffer or PCR machine can result in loci running at different sizes) and experience of user; High, analysis can be impacted by high sequencing error rates, but genotype calls are not influenced by user.

**Box 1: Determining genetic and genotypic diversity of coral hosts and symbionts**

**Figure 3:**
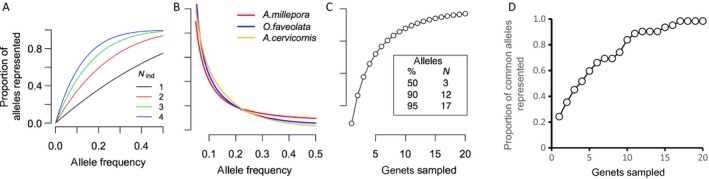
Capturing allelic diversity for coral conservation. Sampling of relatively few genets from a population is sufficient to capture most common alleles. (A) Proportion of alleles observed when sampling a certain number of genets (*N*_ind_) from a population, depending on the allele frequency. For an allele of frequency *P*, the probability of not observing it among *N* diploid genets is (1 − *P*)^2*N*^. Hence, the probability of observing such an allele, which is the same as the proportion of such alleles across the whole genome that are observed, is 1 − (1 − *P*)^2*N*^. (B) The proportion of common alleles (allele frequency *P *>* *0.05) depends on their allele frequency in three species of reef‐building corals and can be calculated based on single nucleotide polymorphism (SNP) data. The three species (*Acropora millepora*,* Orbicella faveolata*, and *Acropora cervicornus*) have broadly similar distributions of their common alleles, despite substantial phylogenetic and geographic separation. (C) Proportion of common SNP alleles represented within a certain number of sampled genets. A sample of just three genets captures >50% of all SNP alleles found in more than 5% of all genomes in the population, and 12 genets capture 90% of them. (D) Proportion of common alleles represented calculated based on microsatellite loci for *Acropora palmata*. Note that due to higher mutation rate of microsatellites they require four rather than three genets to represent >50% of alleles.

Coral host genotypic diversity: In this review, we discuss the importance of both genotypic and genetic diversity on restoration decisions. A “genet” is defined as a genetically unique colony or collection of colonies (“ramets”) that can trace their ancestry back to the same sexual reproductive event (i.e., they stem from the same settler and, hence, share the same genome). Genotypic diversity is the total number of genetically distinct individuals (genets) within a population, whereas genetic diversity is the amount of variation between genotypes on the level of individual genes (Fig. 3). To track the genotypic diversity of corals and their symbionts, it is necessary to determine their unique multilocus genotypes (MLGs) via genetic analysis (Appendix S3: Section 1). While various genotyping approaches have been developed for many of the Caribbean coral species, including allozymes, amplification fragment length polymorphisms (AFLP), microsatellites, and single nucleotide polymorphisms (SNPs; Table 1), the latter two are more routinely used in conservation genetics today (Puckett 2017). Furthermore, microsatellite and SNP loci provide higher allelic variation that can be used to discriminate colonies that share a MLG because they were generated via asexual fragmentation versus those colonies that share an MLG because they are closely related (such as siblings).

Tracking symbiont community diversity is also recommended for fragments and reasonably sized juveniles when possible (see Role of Symbionts). The resolution of Symbiodiniaceae genotypic diversity is constrained by the genetic tools available. The standard internal transcribed spacer 2 (ITS2; LaJeunesse 2001) and chloroplast 23S (cp23S; Santos et al. 2002) rDNA markers are usually sufficient to resolve symbionts to approximately the species level. High‐throughput methods for ITS2 have been developed (Arif et al. 2014, Quigley et al. 2014, Thomas et al. 2018, Smith et al. 2017), although they inevitably underestimate diversity because ITS2 does not resolve all Symbiodiniaceae species (Parkinson et al. 2015b). Methods thus need to be adjusted depending on whether a particular colony is expected to host only one numerically dominant symbiont species (in which case direct sequencing is appropriate) or if it hosts multiple co‐dominant symbiont species (in which case denaturing gradient gel electrophoresis or high‐throughput amplicon sequencing are required; Pochon et al. 2018). Finer‐scale resolution of symbiont diversity at the sub‐species level could be provided by the *psbA* minicircle non‐coding region (Moore et al. 2003, Barbrook et al. 2006, LaJeunesse and Thornhill 2011), microsatellites (Table 1), or SNP markers (currently under development).

Genotyping technology: With the increasing scale of restoration activities, genotyping efforts need to be simplified at minimal costs (Appendix S3: Section 1). Technological advancements in high‐throughput SNP‐based methods such as genotype‐by‐sequencing (GBS) and reduced representation sequencing methods (collectively called RAD‐seq) have made it possible to assess genetic diversity at a large number of single nucleotide variant (SNV) loci for a reasonable cost (Altshuler et al. 2000). However, there is no guarantee that the same set of SNV loci is recovered from each sample in a run or between runs, making clone identification more challenging. Other SNP‐based methods using standardized markers, such as target‐enrichment capture, RAD capture (Hoffberg et al. 2016), microarrays, and microfluidics, also provide information on the order of 10^2^–10^5^ of variants reproducibly (Table 2; Dixon et al. 2016). Regardless of which new approach is chosen, all these methods present additional hidden challenges, requiring advanced bioinformatic training, computational infrastructure, and increased data storage (Table 2). Because resource allocation is an important consideration for practitioners when selecting a genotyping method, we consider two options in depth for obtaining coral MLGs based on the availability of common laboratory equipment, computational resources, and overall budget (Appendix S3: Section S1).

## Choosing Coral Colonies for Restoration: Who and From Where?

Active restoration of the major Caribbean reef builders, specifically the acroporids and the orbicellids, is well underway, and restoration practitioners have already chosen many genets for propagation and outplanting. The classical precept of *primum non nocere* (“first, do no harm”) has traditionally been translated into precautionary concerns about genetic risks, especially preventing genetic swamping or outbreeding depression from affecting the integrity of local populations (Edmands 2007). This premise underlies why the sourcing of restoration material from local areas has been preferred traditionally. This “local is best” (LIB) provenance strategy is sometimes operationally regulated (Tringali et al. 2018), placing strict limits on the geographic range of sourcing corals. However, the poor performance of most local coral populations in recent years suggests that whatever local adaptation is present in these populations may not be adequate to assure persistence as environments continue to change (Williams et al. 2015, *b*, Chan et al. 2019). Hence, we advise considering more flexible provenancing strategies.

Plant restoration planners have similarly articulated provenance strategies that may improve the prospects for climate adaptation in restored populations (Sgrò et al. 2011, Williams et al. 2008 *a*, Prober et al. 2015, Espeland et al. 2017). The simple approach of selecting warmer adapted genets assumes that predictions of environmental conditions are known and that there are no ecological trade‐offs in phenotypes (e.g., poor reproductive performance or poor disease resistance in heat‐tolerant genets). Hence, a so‐called “climate‐adjusted provenance” strategy (sensu Prober et al. 2015) is favored, which incorporates representation of both local genets and genets from across an environmental gradient skewed toward those coming from populations already experiencing predicted future environmental conditions (Fig. 1).

### Assisted gene flow

A climate‐adjusted provenance strategy is recommended when designing assisted gene flow (AGF) interventions. Such AGF interventions recognize that local adaptation is a characteristic of local populations that can be used to improve the fitness of distant populations, especially in scenarios of rapid environmental change. Deliberate translocation of organisms, propagules, or genes among populations can be an effective means to facilitate the spread of adaptive alleles, particularly in areas where populations have declined to the point where colony density or genetic diversity may be insufficient to support successful sexual production of offspring (e.g., where only one known genet persists, (Baums et al. 2006, 2014b). Foundation species with wide ranges and large population sizes can be particularly good candidates for AGF to yield ecosystem‐scale benefits (Aitken and Whitlock 2013). Many coral species fit this profile, and AGF was suggested as a viable management strategy for corals as long as a decade ago (Hoegh‐Guldberg et al. 2008, Riegl et al. 2011).

Embracing AGF interventions has been suggested by several studies (Hoegh‐Guldberg et al. 2008, Dixon et al. 2015, Matz et al. 2018) because corals adapt to local temperature conditions at the genetic level (Polato et al. 2010, Bay and Palumbi 2014, Palumbi et al. 2014, Dixon et al. 2015) and therefore genetic variants conferring heat tolerance should already be present at high frequencies in warm‐adapted populations. In some cases, natural migration appears to be sufficient to exchange these adaptive genetic variants among thermal environments (Matz et al. 2018). Nevertheless, assisted translocation could help ensure such exchange, especially because warming ocean temperatures are expected to change dispersal patterns by shortening planktonic periods and altering current speeds and direction (Heyward and Negri 2010, Baums et al. 2013, Figueiredo et al. 2014, Wood et al. 2006). Models based on population genetic data from *Acropora millepora*, a common coral on the Great Barrier Reef, show that even when immigrants account for as much as 1–3% of the total population there is still no risk of “migrational meltdown” (a reduction of fitness of local population because of an influx of maladaptive alleles) (Matz et al. 2018). This suggests that human‐assisted migration would not pose risks for coral populations still numbering in the tens of thousands, although this may become an issue for populations that have experienced severe declines, such as those in the Florida Keys. Another consideration is the possible limiting effects of cold tolerance when out planting corals sourced from warner environments to colder ones (Howells et al. 2013).

### Nursery and breeding stock selection

In situ nurseries have become an important source for coral stock used in outplanting and breeding efforts. One goal of a nursery should be to ensure good representation of adaptive alleles that are beneficial in various present‐day reef environments, encompassing a sizeable portion of adaptive genetic diversity existing within the species. At this time, we rely on only a handful of traits to identify resilient coral colonies that may carry potentially adaptive alleles, we do not know the identity of the potentially adaptive alleles, and we have very few reliable biomarkers (Box 2). Therefore, we propose the following strategies to maximize the chance that nursery collections will capture alleles that help coral species adapt to various environments (Fig. 3).

**Box 2: Biomarkers**

Biomarkers have emerged as useful tools in many fields, from plant breeding to human disease prediction. Recently, they have been suggested as attractive candidates for use in coral restoration. In theory, restoration practitioners would use a simple bioassay to uncover information about coral colony performance. This information could then be applied to select colonies for parental stocks in larval propagation, rearing in nurseries, or matching outplants to particular sites in anticipation of future environmental challenges. However, biomarkers are often context‐ and species‐specific (Parkinson et al. 2018a), and therefore multiple markers may be needed depending on the types of information desired. In addition, there are many steps between identifying a potential biomarker and deploying it in the field, and the intermediate steps involved in biomarker development are often overlooked. The process involves four major phases: discovery, validation, field trials, and implementation. At present, the majority of basic scientific research has not progressed past the discovery phase (Parkinson et al. 2018a). Consequently, the costs associated with developing practical biomarkers as predictive tools for selective restoration may be high. While the potential savings in terms of time and effort may justify the initial investment, a cost‐benefit analysis must be considered when prioritizing funding for further research in this area at this time.

1. Identify environmentally diverse source reef patches. Within the largest practical area, identify reef sites that differ in their environmental conditions. Ideally, as part of a climate‐adjusted provenance strategy, some of these sites would feature projected future conditions, such as elevated temperature, more variable temperature regimes, and/or lower aragonite saturation state. Our current understanding is that local adaptation in corals happens most prominently with respect to depth (Bongaerts et al. 2011, Prada and Hellberg 2013, Cohen and Dubinsky 2015). But other factors can play a role in adaptation such as temperature (Howells et al. 2013), especially its daily range (Palumbi et al. 2014, Kenkel et al. 2015a). Other environmental parameters such as pH (Comeau et al. 2014, Schoepf et al. 2017), turbidity (Anthony 2000, Anthony and Fabricius 2000), or levels of inorganic nutrients may also play a role in local adaptation, though more research into these specific factors will be required. Even in the absence of detailed environmental data, distinct reef habitats can be identified as sites hosting noticeably different communities of reef organisms. For the success of the sourcing strategy it is important to sample as widely as possible along environmental gradients, in the same depth range as the target restoration sites. The latter is because corals from deep reef habitats (>15–20 m) may be partially or completely reproductively isolated from shallow populations of the same species (Prada and Hellberg 2013, Serrano et al. 2014, 2016, Bongaerts et al. 2017) and may be unsuitable for propagation in shallow nurseries or for restoration of shallow sites.
2. Select colonies for propagation. Collect three to six coral genets per patch, selecting healthy colonies that show some difference in morphology and/or size. They should be growing some distance apart (>5 m; Baums et al. 2006, Foster et al. 2007) to maximize the chance of sampling distinct genets rather than clonemates (Box 1). Collecting just a few colonies per patch might seem insufficient but just three or four diploid colonies would contain about one‐half of all common alleles (frequency >5%) present in a population (Fig. 3). Thus, sampling of three to four colonies from a patch would capture many, if not most, alleles that are locally adaptive since such alleles are expected to be common within the patch due to natural selection.
3. Monitor, replace, and repeat. Propagate selected colonies in the nursery and monitor their performance (see Phenotypic traits of propagated corals). If some corals show poor fitness in the nursery, replace them with other genets from the same patch or habitat type. Excluding poorly performing genets is unlikely to affect representation of the adaptive genetic diversity in the nursery provided representation of all habitat types is maintained. However, it is possible that local adaptation to some reef environments would prove incompatible with the nursery, which is itself a distinct habitat. Although genetic variants in these corals are still valuable as part of the adaptive genetic diversity of the species, we suggest triaging such cases (i.e., omitting genets that perform poorly in nurseries) or omitting the nursery stage by rearing fragments at the native reef (protocols for this are under development; Fragments of Hope, *personal communication*). If survival and growth is merely suboptimal in nurseries rather than absent, then propagating poorly performing genets in the nursery at a minimal level may be warranted if they are performing particularly well during outplanting and have other desirable traits (O'Donnell et al. 2018).

The resulting minimum number of coral genets for each species propagated in a nursery should be on the order of 20–25 and include representatives from reef sites spanning the full range of environmental variation occupied by the species within the restoration jurisdiction. This number of genets would contain >95% of the common alleles present locally within a species (Fig. 3). This approach still omits most of the genetic diversity existing within a species simply because the clear majority of natural genetic variants are rare. In fact, by far the most common type of genetic variant is a singleton, an allele present in a single genet of the species. However, to capture these would require sampling essentially all genets in a species, an approach that is not practical. Rare alleles are by no means useless; while they might not be adaptive at present (given their low abundance) they might become adaptive in the future, when conditions change. Indeed, long‐term adaptation will eventually require replacement of present‐day common adaptive alleles with new ones that are currently rare or even non‐existent (requiring a new mutation to arise). Despite this, comprehensive sampling of alleles that are currently common serves a very important purpose: it gives the restored population a better chance of surviving in the immediate future. With climate‐adjusted provenancing, survival trajectories might be extended by several decades. The longer‐term survival of a restored population will only be ensured if gene flow occurs with adjacent populations (introducing immigrants from other populations that bear novel adaptive alleles), or if adaptive mutations occur within the restored population.

### Sexual propagation and selection of donor colonies

The short‐term adaptive response based on common alleles outlined in the previous section can only happen if outplanted genets reproduce sexually to generate novel allele combinations. Without offspring of such crosses recruiting back to the restored reef, the scope for natural selection cannot extend beyond the originally outplanted genets, and no further adaptation would be possible (barring somatic mutations and genotypic mosaicism; Van Oppen et al. 2015). In this regard, outplanting hundreds or thousands of lab‐reared, sexually produced offspring instead of (or in addition to) adult coral fragments is promising because it would accelerate the process of genetic restoration by one generation. Outplanting sexually produced offspring would be especially valuable for species experiencing long‐term failure of natural recruitment, such as the Caribbean acroporids and orbicellids. Currently in the western Atlantic, gametes for larval production are harvested from wild populations, as well as from fragments in nurseries, via colony netting and subsequent ex situ fertilization in culture vessels. Larval offspring are eventually returned to the field at various stages of development. Sexual propagation and selection of donor colonies should be based on the following strategies.

#### Maximize fertilization success

Most major reef‐building coral species are broadcast spawners that are highly outbred and genetically diverse (Baums 2008, Baird et al. 2009). Therefore, it is usually not necessary to determine their relatedness to avoid inbreeding because the likelihood of picking two parents in natural populations that are closely related as a result of *sexual* reproduction (i.e., are siblings) is vanishingly low (Fig. 2). It must be noted, however, that some Caribbean reef builders (*Acropora* spp., *Orbicella* spp., *D. cylindrus*) can be highly clonal (Fig. 2), where neighboring colonies are genetically identical due to *asexual* fragmentation (Baums et al. 2006, Foster et al. 2007, Miller et al. 2017). These species are hermaphroditic, but are practically self‐incompatible, and consequently successful sexual reproduction requires gametes to be available from (at least) two different genets (Fogarty et al. 2012, Baums et al. 2013). To minimize the chance of harvesting gametes from the same genet, it can be helpful to sample colonies that are relatively far apart (>5 m) (Baums et al. 2006, Foster et al. 2013). The only definitive solution to the clonality problem is to genotype sampled corals (Box 1), which is highly recommended if the budget allows (Appendix S1–S3). Because broadcast‐spawning species typically acquire their algal symbionts from the environment horizontally each generation, effects of breeding design on the population structure and diversity of these symbionts are not a major concern (however, the situation may be different for species that transmit their Symbiodiniaceae from parent to offspring vertically each generation; see Role of Symbionts). In some regions, sexual compatibility can be surprisingly low among conspecific Caribbean acroporids and orbicellids (Fogarty et al. 2012, Baums et al. 2013, Miller et al. 2017), for unknown reasons. For example, there are no data supporting the notion that corals from contrasting environments have lower cross‐fertilization rates. Dixon et al. (2015) crossed colonies from very different thermal regimes and did not observe any differences in fertilization rates (it was nearly 100% for both between‐ and within‐population crosses). In addition, the typical lack of genetic structure across environmental gradients for broadcast‐spawning corals speaks of the absence of strong reproductive barriers imposed by environmental differences (Ayre and Hughes 2000, Baums et al. 2005b, Davies et al. 2015b), except across large depth gradients (Prada and Hellberg 2013). Whether sexual compatibility is determined entirely by genetics or is also context‐dependent remains an open question. Nevertheless, because even a modest percentage of unfertilized eggs in larval cultures can cause significant overall mortality (Pollock et al. 2017), the fertilization success of a coral genet should be monitored whenever possible to improve breeding outcomes. Fertilization success between two‐parent crosses is easily measured by counting the proportion of cleaving eggs two hours post‐fertilization, whereas measuring fertilization success of single parental genets in batch cultures requires genotyping of offspring (Baums et al. 2013, Davies et al. 2015a). Fertilization success is highly dependent on gamete concentration (Oliver and Babcock 1992, Levitan et al. 2004) with optimum in the range of 10^6^ cells/mL. Because, fertilization also declines with gamete age (Oliver and Babcock 1992, Fogarty et al. 2012), the recommended strategy is to use fresh gametes whenever possible. Maximizing parental diversity of batch cultures (more than two parental donors) is the next most important goal. Detailed instructions on how to increase larval survival rates are available from The Nature Conservancy (2018).

#### Use larval crosses to increase local genetic diversity or sexual recruit numbers

During spawning, gamete output is often disproportionate, where one genet provides more spawn than other parents. When designing batch cultures, considerations about the combination of parents and the relative contribution of each parent to the batch will depend on the goal of the breeding program. If the goal is to maximize the genetic diversity of offspring, it is important to ensure equal contribution of parents to the batch culture. However, if the goal is to create the largest number of larvae regardless of their genetic diversity, then all gametes should be added to the culture. We propose that batch cultures for restoration should include at least six parents (Iwao et al. 2014), and more when possible.

#### Maximize settler survival

Survival of outplanted recruits is currently low and presents a bottleneck to the success of sexual recruitment strategies. However, examples of sexual recruits that have survived to sexual maturity and now spawn predictably each year exist (Guest et al. 2014, Chamberland et al. 2016, dela Cruz and Harrison 2017). Settlers survive better when competition with benthic macro‐ and turf algae is low and predation pressure from coral predators is reduced (e.g., *Hermodice carunculata*,* Coralliophila abbreviata*), thus careful site selection for outplanting is paramount (see Outplanting strategies).

#### Enhance adaptation potential via assisted gene flow

Crossing corals and outplanting offspring may be a particularly effective means of AGF. Donor colonies sourced from widely separated and environmentally divergent reefs might be expected to show low survivorship if directly transplanted from one site to another. In contrast, their first‐generation offspring would be expected to show greater phenotypic variation, allowing at least some of them to thrive in either parental habitat. In corals, non‐heritable maternal effects can also have an important impact on the physiology and stress tolerance of early life cycle stages (Dixon et al. 2015, Kenkel et al. 2015b). This can potentially be harnessed to further improve the chance of survival of first‐generation offspring upon transplantation: whenever possible, mothers (i.e., egg donor colonies) should come from the habitat into which the first‐generation offspring are going to be outplanted. While the occurrence of outbreeding depression has never been directly tested in corals, observations in other animals as well as plant species indicate that the risk of outbreeding depression is likely low and should not prevent coral restoration action, especially in local populations that are rapidly declining (Ralls et al. 2018).

#### Interspecies hybridization

Scleractinian corals have a long history of interspecies hybridization (Veron 2011). General evolutionary principles dictate that the long‐term fitness of hybrids must be lower than the fitness of the “purebred” species, otherwise the species would have merged and would not exist as separate units. However, if hybrids can successfully back‐cross (reproduce with the purebreds), they could provide a way to exchange adaptive genetic variation among constituent species. Hybrid corals occur naturally in the Indo‐Pacific as well as the Caribbean (Willis et al. 2014b). In Caribbean acroporids, hybrids between *A. palmata* and *A. cervicornis* exhibit a wide range of morphological variation, and are often found in very shallow, high light environments (Vollmer and Palumbi 1995, Fogarty 2012). As a source for novel morphological diversity, hybridization is an attractive restoration target to enhance reef habitat structure (though clearly not for species recovery). There is little concern about genetic swamping of parental species with hybrid alleles: although these hybrids produce viable gametes (Fogarty 2012), later generation genets are still rare in the population and both parental species have very low rates of successful natural sexual reproduction. In the *Orbicella* species complex, interspecies hybrids are also observed but occur at different frequencies across the Caribbean and perhaps over depth gradients (Fukami et al. 2004). In both hybrid complexes, the hybrid phenotypes may be useful for unique restoration applications such as restoring reef structure in deep, very shallow, or sheltered habitats. Additionally, preliminary lab studies have verified that hybrid *Acropora* offspring can show improved performance in exposure to elevated temperature and CO_2_ conditions. The limited space in nurseries may dictate that practitioners prioritize rearing purebred parents, but at least for sexual larval production, gametes of opportunity may be used to generate interspecies crosses. The outplanting strategy for these hybrid settlers should follow the same recommendations as for the purebred parents (see Outplanting Strategies).

### Phenotypic traits of propagated corals

Given the lack of predictive tools for selecting genets a priori (Box 2), genets will likely perform differently during the propagation phase than they do on the native reef. Performance depends on interactions between genets and their local environment that are difficult to predict (Lirman et al. 2014b, Drury et al. 2017, O'Donnell et al. 2018) and trade‐offs between desirable traits, such as growth and thermal tolerance, may occur (Ladd et al. 2017). Thus, maintaining phenotypic diversity in outplant populations is essential. To that end, tracking key traits in nursery populations will not only help restoration practitioners optimize nursery stocks but can ensure that a diverse suite of potentially important traits is included in outplanting designs. However, traits are not all equally relevant to success and measuring some traits can be challenging, time consuming, and cost prohibitive, diverting resources from essential tasks such as outplanting and nursery maintenance. Therefore, establishing consistent practical guidelines for collecting the most informative data will be important to optimally manage nurseries.

While most genets can survive and propagate in nursery conditions, there are often more genets available within a nursery's area of interest than can be maintained. When this is the case, traits that can help guide which genets to propagate are (1) partial mortality, (2) rate of wound healing, (3) skeletal growth rate, (4) bleaching and infectious disease resistance or resilience, and (5) sexual reproductive output (Table 3).

**Table 3:** Phenotypic traits that can help in selecting coral genets for propagation and restoration

Trait	Measurement
Partial mortality	amount of tissue loss
Wound healing rate	days to heal from fragmentation
Skeletal growth rate	buoyant weight or “crown area”
Bleaching and infectious disease resistance/resilience	no bleaching/infectious disease or recovers quickly
Sexual reproduction output	spawning and sperm motility

#### Partial mortality

Partial mortality is common in coral colonies due to a variety of impacts, including corallivory, competition, fragmentation, and temperature stress (Lirman et al. 2014a). Partial mortality can lead to increased susceptibility to infectious diseases. Thus, percent recent mortality is a reliable indicator of recent stress across a range of coral species (Cooper et al. 2009, Lirman et al. 2014a) and can be visually estimated directly or from photographs.

#### Wound healing rate

Corals that heal rapidly after sustaining lesions or fragmentation have a lower probability of secondary infection (Palmer et al. 2011) and therefore higher probability of survival. Furthermore, healing rate may be indicative of improved physiological condition (Fisher et al. 2007) and/or greater energy reserves (Denis et al. 2011), which can help corals survive bleaching events (Rodrigues and Grottoli 2007, Levas et al. 2013, 2018, Grottoli et al. 2014, Camp et al. 2018).

#### Skeletal growth rate

Corals that grow rapidly in nurseries (i.e., high calcification rate) can be fragmented more frequently to produce more stock. Corals that grow rapidly in the field generate more structural complexity, which is often a primary goal of reef restoration. Unfortunately, linear extension rate (a frequently applied measure of coral growth) is not always correlated in the nursery and the field (O'Donnell et al. 2018) and growth rates are often not correlated with survival (Ladd et al. 2017). However, there is evidence that calcification rate may be genetically influenced and therefore represents a good measure of genet performance, whereas linear extension rate and skeletal density vary with environmental conditions (Kuffner et al. 2017). Although quantifying calcification rate by measuring buoyant weight is time‐consuming (Jokiel et al. 1978, Herler and Dirnwöber 2011, Morrison et al. 2013), investing the time and resources initially to quantify calcification in this way when selecting genets could have long‐term payoffs in nursery and outplanting efficacy. However, for tracking genet growth in the field, we suggest a metric commonly used in silviculture termed the “crown area” (CA) of a tree (Uzoh and Ritchie 2007). Crown area defines the two‐dimensional footprint under the tree canopy and, thus, would work for branching and massive coral morphologies alike, and represents the coral's capacity for harvesting direct, downwelling solar irradiance. The measurements needed to calculate crown area are the long width of the crown (CWL) and the short width of the crown (CWS, perpendicular to the long width), both of which can be easily estimated using a tailor's tape, and the area of the footprint calculated using the formula

CA=CWL×CWS×π/4.

In addition to yielding important information on how much reef‐substratum area each coral colony claims per unit of time, it is a simple and straightforward measurement of planar coral‐surface area with which to normalize calcification rates (Kuffner et al. 2013) or other physiological measurements.

#### Bleaching and disease resistance and/or resilience

Threats to coral reefs are numerous but disease and bleaching are two of the main drivers of coral decline in the wild (Halpern et al. 2008, Harborne et al. 2017) and in restoration projects (Drury et al. 2017, Ladd et al. 2017). Importantly, infectious disease prevalence and the frequency and severity of bleaching events are expected to increase over the coming century (Miller et al. 2014, Maynard et al. 2015, van Hooidonk et al. 1996) and bleached corals are often less resistant to infectious diseases (Muller et al. 2018). Therefore, to maximize the chances of successful restoration outcomes, nurseries should expend efforts to identify and maintain genets that are resistant or resilient to thermal stress and disease (i.e., do not bleach and do not contract infectious disease, or rapidly recover from both). Although multiple traits can impart resilience to one or both of these stressors, such as the identity and diversity of algal symbionts, heterotrophic capacity, energy reserves, and environmental history (Baker et al. 2004, Berkelmans and van Oppen 2006, Anthony et al. 2009, Hughes and Grottoli 2013), many of these traits are currently time‐consuming or difficult to measure, and require specialized training and equipment. Instead, restoration practitioners should focus on simple, qualitative surveys to track the prevalence of mortality, bleaching, and infectious diseases among genets. Survey data should be collected at least twice a year to coincide with the timing of bleaching and/or infectious disease outbreaks in that region (although more frequent surveys are desirable). Surveys should involve recording the number and proportion of ramets from each genet experiencing bleaching or disease and whether the recorded stressor resulted in partial or complete ramet mortality, or if the ramet survived (Muller et al. 2018). Genets that show high mortality due to infectious disease or bleaching should be replaced within the nursery, preferably from the same source site and from parent colonies that appear to also have recovered from the stress or were less susceptible to the stress.

#### Sexual reproduction

Fecundity is a necessary, but insufficient, trait to assure reproductive success given the requirements for spawning synchrony and fertilization compatibility (see Sexual propagation and selection of donor colonies). The cultivation of large adult fragments as “spawning stocks” within nurseries can provide opportunities to monitor the presence/absence of spawning and synchrony in spawn time among genets as well as to collect and rear sexual offspring. Reproductive size will vary considerably among species (Soong and Lang 1992) so maintaining multiple colonies of all genets above spawning size in nurseries may be more feasible for some species than others. Genets with low reproductive output that are low performing in the other phenotypic trait categories should be reevaluated as candidates for propagation stocks. A low‐cost method to assess gamete quality is to observe sperm motility (Hagedorn et al. 2006), which is highly predictive of fertilization success. Sperm motility can be scored by observing live sperm through a low‐end phase contrast microscope and estimating the proportion of sperm that are moving (M. Hagedorn, *personal communication*). This is cheaper and faster than assessing egg quality (e.g., by analyzing their lipid content). Sperm motility is likely influenced by genetic factors as well as environmental factors (such as heat stress (Hagedorn et al. 2016)) and is consequently a good measure of genet performance in a given environment.

## Outplanting Strategies

The selection of sites to establish restored populations is a strong factor in the ultimate success of outplants. Many previous outplanting efforts show high variation in survivorship among sites that are not easily attributable to specific ecological drivers (Bowden‐Kerby 2008, Bowden‐Kerby and Carne 2012, Lirman et al. 2014b, Drury et al. 2017). Potential contributing factors include protection status, visitation, intact trophic structure of the reef (including herbivory and corallivory levels), water quality, historical occurrence of the species, and alleviation of the stressors that caused the original coral decline (The Nature Conservancy checklist; *available online*).^11^ Individual species need to be outplanted to sites within their general habitat niche (e.g., *A. palmata* prefers relatively shallow, high energy habitats) but more specific guidelines can be difficult to establish due to the lack of baseline data, species‐specific differences in niches (Goreau 1959, Dollar 1982), and uncertainty as to how those niches may change in the future. Historical and fossil records may be used as guides to where species flourished recently and during the warmer mid‐Holocene, providing insight for restoration siting (Toth et al. 2017).

#### Site selection for fragments

Within species, there is an expectation that genets should survive better in a habitat similar to their native habitat. Direct reciprocal transplant experiments with various species support this expectation (Howells et al. 2013, Kenkel et al. 2013). However, studies examining habitat‐specific performance of nursery‐reared *Acropora* spp. do not show patterns of preferential performance (e.g., in linear growth or mortality) in native sites or habitat types (Drury et al. 2017, O'Donnell et al. 2018, Pausch et al. 2018). There are various reasons this expectation may not play out, such as a lack of local adaptation in the first place, or acclimation during the nursery propagation period that decouple this affinity. Stochastic disturbance as well as rapid change in environmental conditions within individual sites may also exacerbate the mismatch of genets with native habitats. In the absence of better predictive capacity in matching genet performance (see Box 2), our overall recommendation is to adopt a similar climate‐adjusted provenance strategy in outplanting genets among individual sites (Fig. 1), with proportional representation of all genets from available stocks outplanted among sites.

#### Increasing potential for sexual reproduction

To maximize the potential for sexual reproduction in the restored population of broadcast‐spawning species, more than one genet is required. Two genets may provide only marginal fertilization potential for outcrossing hermaphrodites, while mixtures of four to six parents showed high fertilization success in *Acropora* spp. (Baums et al. 2013, Iwao et al. 2014, Miller et al. 2017). Higher numbers of parental genets (~10) are likely needed for gonochoristic species because sex ratios are often skewed, or for *Acropora* spp. due to lack of synchrony during spawning and variability in fertility among genets (Fogarty et al. 2012, Miller et al. 2016). Hence, we recommend mixtures of 5–10 genets for batch culturing of all species. Optimal distance between outplants is unknown but 2–3 m may work well for fast growing acroporids and orbicellids (not too far for successful fertilization, not too close that colonies compete for resources). If more genets are available in nursery stocks, they can be allocated among sites in different combinations or, if phenotypic information on stress‐resistant genets is available, they may be stratified among sites to ensure that some resistant phenotypes are outplanted to each site. In special cases where certain genets display multiple positive traits (e.g., low partial mortality and high skeletal growth rate), these “winners” may be included at as many sites as feasible, keeping in mind that these nursery “winners” may not necessarily exhibit superior performance in the wild.

#### Site selection for sexual recruits

In the case of restoration based on sexual reproduction, each juvenile represents a unique genet. Thus, the number of genets outplanted to a site will be dramatically higher than can be achieved when outplanting nursery‐reared coral fragments. We propose that similar strategies in site selection be employed for the outplanting of sexual recruits as for fragments. The goal is to mix and match as many genets as possible to maximize genetic diversity, which is the basis of adaptive resilience (Hermisson and Pennings 2005, Baums 2008, Whiteley et al. 2011).

#### Monitoring

It is too time consuming and challenging to monitor very small sexual propagules, but as recruit sizes increases, they are relatively easy to track. Subsequent monitoring of the success of genets within each outplant site can provide important insights into their performance. Where tracking the phenotypic performance of genets across sites is feasible, resulting data should be contributed to genet/phenotype databases (see *Future research needs*). In the simpler case where the fate of genets is not tracked, colonies that expire should be replaced with fragments from genets that were not originally included at that site.

## Role of Symbionts

### Role of symbiont in an asexual restoration program

Reef corals are mutualistic symbioses between diverse and taxonomically divergent taxa that collectively comprise the coral “holobiont” (Rohwer et al. 2001), and the identity of these partners can have important effects on the phenotypic characteristics of coral colonies (Baker 2003). In particular, diversity in the algal symbiont community (family Symbiodiniaceae) has been linked to variation in holobiont thermotolerance (Glynn et al. 2001, Baker et al. 2004, Berkelmans and van Oppen 2006, LaJeunesse et al. 2010), growth rate (Jones et al. 2008, Cunning et al. 2015, Pettay et al. 2015), irradiance optima (Rowan and Knowlton 1995, Rowan et al. 1997, Iglesias‐Prieto et al. 2004), and possibly disease resistance (Correa et al. 2009), all of which are relevant to restoration objectives. Thus, algal symbionts may affect restoration outcomes, though evidence for such impacts in nurseries or outplant sites is currently lacking in the few cases where they were investigated (Lirman et al. 2014b, Parkinson and Baums 2014, O'Donnell et al. 2018, Parkinson et al. 2018b).

Because many coral species commonly used for restoration are capable of hosting several species and strains of Symbiodiniaceae, at least when sampled across their biogeographic range, algal symbiont diversity may create phenotypic and genetic differences among restoration units that are not attributable to the coral host (Parkinson and Baums 2014). However, unlike host genetic differences, symbiont genetic differences may not be fixed over a coral genet's lifespan, as the symbiont community can potentially change over time in response to disturbances and/or prevailing environmental conditions. However, barring direct collaboration with scientists to identify and monitor changes in symbiont communities using genetic methods, it may not be practical for most restoration practitioners to take explicit account of Symbiodiniaceae diversity for the time being. Although an active area of research, prescriptive methods of manipulating symbionts as part of routine nursery propagation and outplanting have yet to be identified, and the longevity of these manipulations has yet to be established in the field (Peixoto et al. 2017). At present, it should be sufficient for practitioners to realize that exposure of nursery‐grown corals to specific environmental conditions might lead to changes in symbiont communities, leading to variation in outplanting success. Climate‐adjusted provenancing might represent a pragmatic approach to dealing with this uncertainty. Warmer local environments are expected to have a higher abundance of both thermotolerant coral host genets and thermotolerant symbionts, so sourcing holobionts from such locations may help outplanted corals survive long‐term under climate change conditions.

### Role of symbionts in a sexual restoration program

An important question in sexual propagation is *when* (i.e., at what age) to outplant sexual offspring. Evidence suggests that increasing recruit size increases outplant survivorship (Raymundo and Maypa 2004) such that an extended grow‐out period in the nursery may be worthwhile provided costs of ex situ rearing can be minimized. For species with larvae that lack symbionts (i.e., those that acquire symbionts via horizontal transmission), the process of symbiont colonization during early life deserves attention. The onset of symbiosis can be as early as 2–6 days after fertilization (Schwarz et al. 1999, Edmunds et al. 2005, Harii et al. 2009). In rare cases, the association is highly specific from the outset, where the host harbors one algal species from juvenile to adult stages (e.g., *Fungia scutaria*; Weis et al. 1992). More commonly, during early onset the association is plastic (with the formation of partnerships sometimes involving multiple symbiont species), but ultimately shifts to a stable dominant species of Symbiodiniaceae during the juvenile stage (Coffroth et al. 2001, van Oppen 2016, Abrego et al. 2009, Cumbo et al. 2013, Yamashita et al. 2013). While colonization itself does not seem to be a limiting step, symbiont community composition is another major determinant (along with host genet) of physiological performance of the coral (Little et al. 2004, Parkinson et al. 2015a, Grottoli et al. 2018) and is expected to vary with outplant environment.

#### Provisioning of sexual recruits

In the natural environment, typical Symbiodiniaceae sources for coral recruits include the water column, sediments, and adult corals (Coffroth et al. 2006, Adams et al. 2009, Nitschke et al. 2016, Cunning et al. 2017, Ali et al. 2018, Quigley et al. 2018). It is straightforward to provision coral juveniles with mixtures of cultivated micro‐algae when they are kept in small aquaria ex situ, but only a subset of the Symbiodiniaceae diversity is currently cultured. Further, relatively little is known regarding how to provision particular symbionts, in situ*,* in such a way as to maximize their uptake by coral larvae or juveniles. Environmental conditions affect larval symbiont acquisition, with increasing temperatures appearing to select for thermotolerant symbionts, at least in *Acropora* from the Great Barrier Reef (Abrego et al. 2012). One strategy to facilitate the acquisition of appropriate symbionts is thus to outplant settlers prior to symbiont colonization. That way, Symbiodiniaceae are acquired from the outplant environment, likely promoting symbionts that are specialized for that environment (LaJeunesse et al. 2004, 2010, Abrego et al. 2012, Howells et al. 2012). However, maturation of the symbiont community can take 1.5–3.5 years (Abrego et al. 2009, Yamashita et al. 2016), and does not always match that of the adult community at the outplant site (Little et al. 2004). There is relatively less information on the acquisition of different symbionts by Caribbean scleractinian corals, but laboratory studies suggest that an uptake window of up to 4 months exists (McIlroy and Coffroth 2017), and that it can take up to 4 years before the community stabilizes (Coffroth et al. 2010, Poland and Coffroth 2017). A hybrid approach has been taken by managers and researchers in Mexico who transfer outplants to the reef for two weeks to take up local symbionts and then return them to the nursery to achieve additional growth before final outplanting (Claudia Padilla and Ania Banaszak, *personal communication*). Additional research on the optimal timing of outplanting sexual recruits is needed.

#### Rare species

Rare coral species that also associate with rare symbionts such as the threatened Caribbean pillar coral, *Dendrogyra cylindrus* and its symbiont *Breviolum dendrogyrum* (Lewis et al. 2018, Chan et al. 2019) face additional challenges. Because adult *D. cylindrus* are the only known reservoirs for this symbiont, and these populations are in rapid decline, it cannot be assumed that natural sexual recruits will readily be able to acquire appropriate symbionts. Consequently, larval recruits might have to be exposed to adult conspecifics, or special efforts made to culture these Symbiodiniaceae specifically for restoration.

## Future Research Needs

The recommendations in this review are based on the best available science, but knowledge gaps exist that should be the focus of future research. In general, it has to be recognized that nursery rearing of both larvae and adult fragments imposes unintended selection for nursery‐adapted genets (Christie et al. 2012, 2016, Morvezen et al. 2016, Horreo et al. 2018, O'Donnell et al. 2018), although the strength of the selection is not yet known but could be estimated. For example, larval culturing selects gametes and larvae that can survive netting, high‐density rearing, handling during water changes, settling on artificial substrates, and so on. Data from other systems demonstrate that after removal of initial culture selection, species can adapt to wild conditions rapidly, especially if time spent in captivity is short (Espeland et al. 2017, but see Horreo et al. 2018). Our review already includes recommendations to reduce unintended selection such as diversified provenancing (over different habitats and times), intentionally promoting gene flow, and matching the nursery and reef environments as closely as possible (Frankham 2008, Espeland et al. 2017). Both outbreeding and inbreeding depression have yet to be tested experimentally in corals and should be a focus of future research. Outbreeding depression could occur despite our expectations, especially if the number of outplanted genets approaches the number of genets surviving in the wild. Consequences include (1) wasting resources by transplanting maladapted genets and (2) preventing rapid local adaptation when foreign genotypes reproduce with local genets. We are not aware of definitive evidence for outbreeding depression in corals, but the topic is receiving renewed attention in marine species (Pritchard et al. 2013, Pereira et al. 2014, Ellingson and Krug 2016, Phillips et al. 2017). Inbreeding depression could occur after repeated rounds of sexual reproduction among closely related genets in a nursery setting but that seems a far‐off risk, given the challenges of long‐term coral culture and breeding combined with relatively long generation times.

Unintended introduction of invasive pests when moving corals over large distances is another concern. This risk is particularly relevant for corals given the dire toll infectious disease have had on coral populations (Walton et al. 2002) and lack of screening tools for pathogens. Translocating larvae or gametes (i.e., cryo‐preserved sperm), when undertaking AGF, rather than adult fragments, is a good option to minimize this risk (Hagedorn et al. 2012) and further refinement of these methods is a research priority. Basic biosecurity practices such as visual inspections and quarantine can also reduce risk of unintended pest introduction (IUCN/SSC 2013).

To improve the prospects for leveraging specific genets and traits in future restoration, it is crucial to link coral genets with phenotypic performance and fine‐grain environmental data. We call for an open‐access database to catalog all managed genets in nurseries and outplants. This would also address a specific aim (Action 4a) in the Endangered Species Act recovery plan for *A. palmata* and *A. cervicornis* (NMFS 2015). Achieving this goal will require a coordinated effort by practitioners and researchers to report standardized metadata for each genet in the database allowing integration with other ongoing monitoring efforts (e.g., Atlantic and Gulf Rapid Reef Assessment database; *available online*).^12^ At a minimum, the database should contain information about the collection site (Geographical Positioning System coordinates), collection date, sample depth, and collector information. Additional information on the phenotypic traits and environmental variables discussed should be collected when possible. Finally, if the budget permits genotyping (Box 1), a clonal identification number should be assigned to genets based on the comparison of their multilocus genotype (MLG) pattern against a background of archived genotypes (for example, previously detected acroporid genomic variants; Ktichen et al. 2019).

A way forward is the integration of the recommended actions into a decision‐making framework to decide on how and when interventions should be made, and the development of such a framework is another recommended research priority (Anthony et al. 2015).

## Conclusions

Reef restoration has made significant progress via the development of innovative ways to grow and outplant corals. While the task of restoring reefs in the face of rapid climate change remains daunting and will prove futile if current carbon emissions continue unabated, coral species still harbor a significant amount of genetic variation that can be leveraged to enable rapid adaptation if the restoration community adopts an evolution‐centric strategy. The heterogeneity in response to stress is what provides the hope that reef‐restoration work is worth the time and effort. We posit that this natural heterogeneity can be capitalized upon, using the recommendations herein, in a way that may speed up natural selection and provide a means for certain species and coral ecosystems to persist.

## Notes

11
http://www.reefresilience.org/restoration/project-planning/site-selection

12
http://www.agrra.org/
