# Supplementary material for: Considerations for maximizing the adaptive potential of restored coral populations in the western Atlantic
Source: Ecol Appl. 2019 Aug 19;29(8):e01978. doi: 10.1002/eap.1978 (PMC6916196; doi:10.1002/eap.1978)
Supplement: Supplementary file 1 [file EAP-29-na-s001.pdf]

Supporting Information. Baums, I. B., A. C. Baker, S. Davies, A. G. Grottoli, C. Kenkel, S. A. Kitchen, I. B. Kuffner, T. LaJeunesse, M. Matz, M. Miller, J. Parkinson, and A. A. Shantz. 2019. Considerations for maximizing the adaptive potential of restored coral populations in the western Atlantic. *Ecological Applications*.

Appendix S1: Section 1

Genesis of the review

In response to rapidly deteriorating climate, land, and ocean conditions and the concomitant expansion of active coral restoration in the Caribbean, the Coral Restoration Consortium (CRC) was created in 2017 as a coordinating body that seeks to disseminate best practices, foster technological innovation, and identify key research gaps to improve the efficiency with which coral reefs are restored in the Wider Caribbean. The goal is to restore reef ecosystems so they can protect coastlines, foster fisheries, serve as the basis for many economies, and be enjoyed for their diversity by future generations. The Coral Restoration Consortium's mission is to foster collaboration and technology transfer among coral restoration scientists, practitioners, and managers, and to facilitate a community of practice that will advance the multiple, rapidly changing paths to active coral restoration such that reef restoration keeps pace with rapidly changing ocean and environmental conditions. The Coral Restoration Consortium is driven by action and results. We believe that through innovation, open access to data and education, and a collaborative, coordinated approach, we can provide a strategic plan and supporting resources to restore coral reefs in an ecologically meaningful manner to help ensure their sustainability for future generations. The Working Groups (WG) of the CRC were established to implement the CRC priorities - adapted from the recommendations that arose from the *Workshop to Advance the Science and Practice of Caribbean Coral Restoration* held in

November 2016. The Restoration Genetics Working Group was established to address the following CRC priority:

4. Develop recommendations on several issues related to coral genetics (e.g., guidelines on how to characterize out-planted genotypes, a clearinghouse of genotypic information on outplants, quantitative recommendations on how to maintain and enhance genetic diversity over a given geographic region.)

Participation in the working group is open to anyone interested in coral restoration. An introductory call-in meeting chaired by IB was held in May 2017 followed by quarterly calls. The working group consists of scientists at academic and government institutions, managers, non-for profit groups and private citizens. Its 82 members represent 16 countries. The working group has a self-identified core group (the authors) and a wider group of interested parties that participate in calls but do not contribute to writing or method development. The working group decided to write a synthesis paper to address the priorities outlined above. An outline identifying the topics in need of recommendations from the WG was circulated among the core and wider group for comment. The core working group met in person in May of 2018 to prepare a draft of the evidence synthesis. The core working group considered all published scientific papers relevant to the evolutionary and ecological dynamics of Caribbean reef-building corals by conducting literature searches on the Web of Science and Google Scholar. Evidence was weighted by the study design, number of samples included in the study, the inclusion of appropriate controls and the geographic scale of the study. For example, for us to consider a study as providing evidence for local adaptation, the study had to include a reciprocal transplant experiment. When there were disagreements, the group sought further evidence from the literature and/or analyzed existing data until a consensus was reached. For example, we

disagreed on the number of genotypes necessary to represent the majority of potentially locally adapted alleles. Therefore, we analyzed existing genetic data representing several species and genetic markers to arrive at a number. These analyses were included in the paper. Some recommendations are based on broad evidence while others are not as well supported. We identified these cases in the paper and specified future research needs. We circulated a draft of the synthesis evidence to the wider working and the steering committee of the CRC prior the December 2018 conference "Reef Futures" held in Key Largo, FL and presented our findings at that conference. A keynote presentation to the entire conference was followed by a dedicated session on this topic that included four presentations and an open discussion period. Feedback from the conference participants was incorporated into the synthesis paper.
