# Supplementary material for: Considerations for maximizing the adaptive potential of restored coral populations in the western Atlantic
Source: Ecol Appl. 2019 Aug 19;29(8):e01978. doi: 10.1002/eap.1978 (PMC6916196; doi:10.1002/eap.1978)
Supplement: Supplementary file 2 [file EAP-29-na-s002.pdf]

Supporting Information. Baums, I. B., A. C. Baker, S. Davies, A. G. Grottoli, C. Kenkel, S. A. Kitchen, I. B. Kuffner, T. LaJeunesse, M. Matz, M. Miller, J. Parkinson, and A. A. Shantz. 2019. Considerations for maximizing the adaptive potential of restored coral populations in the western Atlantic. *Ecological Applications*.

Appendix S2

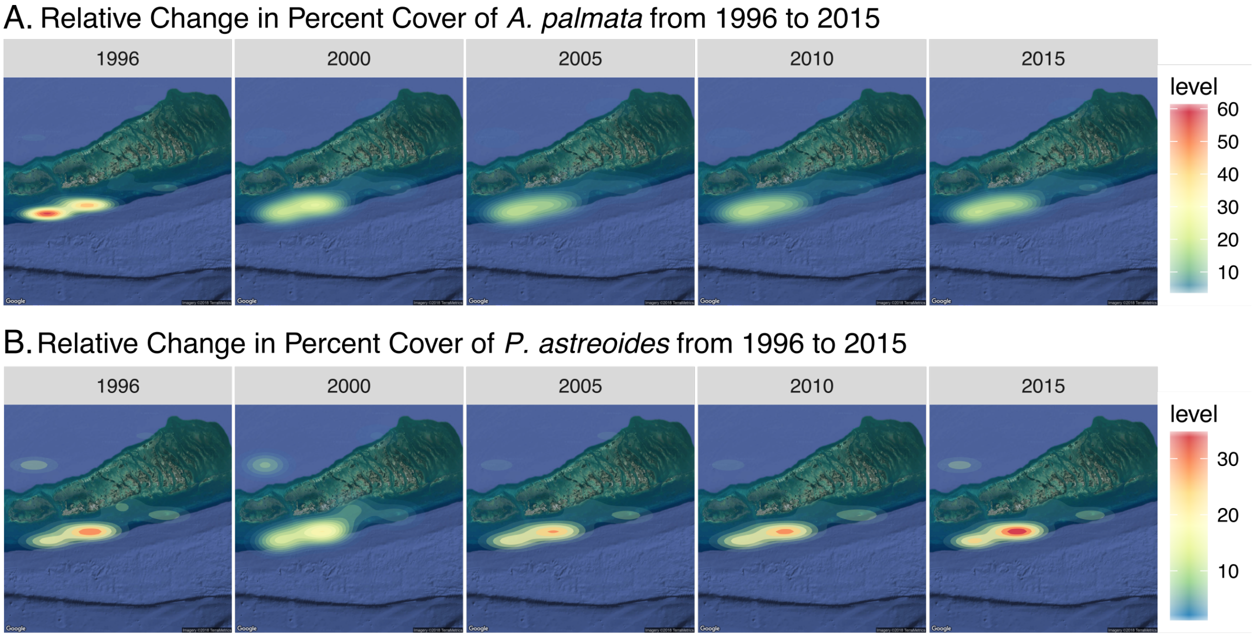

Figure S1. Spatial heatmap of the relative changes in percent coral cover of the major reef-building species, *Acropora palmata* (A) and the weedy brooding species, *Porites asteroides* (B) from 1996 to 2015 in the Lower Florida Keys. Coral cover data was obtained from the fixed CREMP survey stations, omitting sites for which the full 20 years of data were not available. Raw survey data were multiplied by 1000 and log-linear-hybrid transformed and rounded to the nearest whole number. A spatial heatmap was plotted by converting cover to density of particular survey-coordinates and using these as the shading parameter. This visual is meant to serve as a means to assess and re-evaluate restoration needs. For example, (A) *Acropora palmata* is recommended for restoration given the dramatic and persistent decline, while (B) *Porites asteroides* is not recommended at the current time given its stable population size. Species must be re-evaluated regularly to capture changes in status.
