# Supplementary material for: Considerations for maximizing the adaptive potential of restored coral populations in the western Atlantic
Source: Ecol Appl. 2019 Aug 19;29(8):e01978. doi: 10.1002/eap.1978 (PMC6916196; doi:10.1002/eap.1978)
Supplement: Supplementary file 3 [file EAP-29-na-s003.pdf]

Supporting Information. Baums, I. B., A. C. Baker, S. Davies, A. G. Grottoli, C. Kenkel, S. A. Kitchen, I. B. Kuffner, T. LaJeunesse, M. Matz, M. Miller, J. Parkinson, and A. A. Shantz. 2019. Considerations for maximizing the adaptive potential of restored coral populations in the western Atlantic. *Ecological Applications*.

Appendix S3: Section 1

Options for genotyping coral colonies

Option 1: For practitioners with limited or no lab and computational resources

The "genotyping" option requiring the least amount of resources involves grafting live coral fragments to existing colonies to test for tissue rejection between non-clonemates (Neigel and Avise 1983, Resing and Ayre 1985, Jokiel et al. 2013). However, this method has proven unreliable and is not recommended. The only definitive way to identify clones is via screening of genetic markers. Nevertheless, tissue rejection assays might be the most feasible option for practitioners with few resources.

To facilitate the screening of genotypic diversity for a coral species without easy access to laboratory space or a bioinformatician, the best option is to purchase off-the-shelf genotyping products, such as the microarray- and microfluidics-based SNP approaches. Such an off-the shelf solution is now available for the genus *Acropora* from Affymetrix (Kitchen and Baums, in prep). The Affymetrix custom genotyping array is in production for the acroporids with ~35,000 SNP loci identified (~~Ktichen et al. 2018~~). To obtain MLGs, results from the array will be run through a web portal that performs an automated, standardized bioinformatics protocol in R (Team 2013)(Kitchen and Baums, unpublished). The genotype information should ultimately be deposited in a database as described previously (see Future research needs in the main text).While these approaches are more desirable in the long-run, they come with up-front costs for SNP identification and development, which must be performed for every new genus and

ideally for every species by a researcher with access to a molecular laboratory and computational resources before they are available to practitioners. For acroporids and orbicellids, SNP markers identified in previous studies (Drury et al. 2016, Prada et al. 2016, Ktichen et al. 2018) can already be used by researchers to develop such tools. For other species, genomic resources will have to be established, after which the high resolution SNV markers can be analyzed cost-effectively in a standardized manner. These approaches are attractive because sample preparation and processing are completed by the sequencing facilities eliminating the need for laboratory equipment. The resulting raw genotype data can be analyzed using the open-access R packages Poppr (Kamvar et al. 2014) or Rclone (Bailleul et al. 2016) with basic computer specifications to assign MLGs. A standardized bioinformatics pipeline and genotype database requiring no bioinformatics background and only a browser to access it are now available to analyze the Acropora SNP array data (http://coralsnp.vmhost.psu.edu:8080/).

Option 2: For practitioners with laboratory and computational resources

For corals for which there is no genotyping platform such as a SNP-chip, a highly cost-efficient and robust solution to keep track of genets is the method known as reduced-tag-representation-2bRAD (RTR-2bRAD, (Wang et al. 2012)). The 2bRAD method belongs to the family of Restriction site Associated DNA (RAD) sequencing methods and is one of the simplest RAD protocols distinguished by three features that are particularly valuable in the coral restoration context. First, it does not involve size selection of sequenced fragments, which ensures consistency across samples and allows for easy standardization across labs and genotyping rounds. Second, it can be performed on substantially degraded DNA. Third, it interrogates only 1/16th or even 1/64th of all restriction endonuclease recognition sites in the genome, resulting in high sequencing depth per locus at reasonable costs. 2bRAD sequence tags

are limited to 36 bp which, theoretically, may lead to uncertainties when analyzing complex genomes (Andrews et al. 2016). However, in practice 2bRAD tags are in fact long enough to find a unique match even in the human genome (Wang et al. 2012). In corals, 2bRAD provides tens of thousands of variable markers enabling sophisticated demographic modeling (Matz et al. 2018). We have successfully identified clonal and population structure in *Acropora cervicornis*, *Orbicella faveolata*, *Montastrea cavernosa*, and *Siderastrea siderea* using 2bRAD, notwithstanding the fact that all these experiments involved independent library preparations and sequencing runs (M. Matz, unpublished).

While RTR-2bRAD library preparation and sequencing could soon be ordered as a service for under $50/sample, practitioners would still face two challenges: isolating high-quality (although not necessarily high-integrity) DNA from corals and analyzing the resulting sequencing data. DNA isolation procedures for the major Caribbean reef-builders (acroporids and orbicellids) have been already developed (Table 2), but other coral species might require species-specific protocol modifications. The bioinformatic pipeline for 2bRAD data analysis (available at https://github.com/z0on/2bRAD_denovo) requires basic familiarity with command-line Unix environment and R; in our practice, the required training for non-initiated people takes about two days (10-12 hours total). Soon, generic 2bRAD analysis steps to identify clones and analyze population structure will be automated and available as a web-based service.

References

Andrews, K. R., J. M. Good, M. R. Miller, G. Luikart, and P. A. Hohenlohe. 2016. Harnessing the power of RADseq for ecological and evolutionary genomics. Nat Rev Genet **17**:81-92.

Bailleul, D., S. Stoeckel, and S. Arnaud-Haond. 2016. RClone: a package to identify MultiLocus Clonal Lineages and handle clonal data sets in r. Methods in Ecology and Evolution **7**:966-970.

Drury, C., K. E. Dale, J. M. Panlilio, S. V. Miller, D. Lirman, E. A. Larson, E. Bartels, D. L. Crawford, and M. F. Oleksiak. 2016. Genomic variation among populations of threatened coral: *Acropora cervicornis*. Bmc Genomics **17**:286.

Jokiel, P. L., K. S. Rodgers, and S. A. Karl. 2013. Comparison of grafting versus a molecular genetic technique in identifying clone mates of the reef coral *Porites rus*. Marine Ecology Progress Series **477**:87-92.

Kamvar, Z. N., J. F. Tabima, and N. J. Grünwald. 2014. Poppr: an R package for genetic analysis of populations with clonal, partially clonal, and/or sexual reproduction. PeerJ **2**:e281.

~~Ktichen, S., A. Ratan, O. Bedoya-Reina, R. Burhans, N. Fogarty, W. Miller, and I. Baums. 2018. Genomic variants among threatened *Acropora* corals bioRxiv.~~

Matz, M. V., E. A. Treml, G. V. Aglyamova, and L. K. Bay. 2018. Potential and limits for rapid genetic adaptation to warming in a Great Barrier Reef coral. Plos Genetics **14**:19.

Neigel, J. E., and J. C. Avise. 1983. Clonal diversity and population structure in a reef-building coral, *Acropora cervicornis* : Self-recognition analysis and demographic interpretation. Evolution **37**:437-453.

Prada, C., B. Hanna, A. F. Budd, C. M. Woodley, J. Schmutz, J. Grimwood, R. Iglesias-Prieto, J. M. Pandolfi, D. Levitan, K. G. Johnson, N. Knowlton, H. Kitano, M. DeGiorgio, and M. Medina. 2016. Empty Niches after Extinctions Increase Population Sizes of Modern Corals. Current Biology **26**:3190-3194.

Resing, J. M., and D. J. Ayre. 1985. The usefulness of the tissue grafting bioassay as an indicator of clonal identity in scleractinain corals (Great Barrier Reef - Australia). Pages 75-81 *in* Proceedings of the Fifths International Coral Reef Congress, Tahiti, Tahiti.

Team, R. C. 2013. R: A Language and Environment for Statistical Computing. R Foundation for Statistical Computing, Vienna, Austria.

Wang, S., E. Meyer, J. K. McKay, and M. V. Matz. 2012. 2b-RAD: a simple and flexible method for genome-wide genotyping. Nature Methods **9**:808-+.
